# Supplementary material for: Comparative Analysis of Secondary Metabolites in Diplodia corticola Strains with Different Virulence Degrees Associated with Canker and Dieback of Quercus spp
Source: Molecules. 2023 Aug 28;28(17):6302. doi: 10.3390/molecules28176302 (PMC10489718; doi:10.3390/molecules28176302)
Supplement: Supplementary file 1 [file molecules-28-06302-s001.zip › molecules-2532776-supplementary.pdf]

Supplementary Materials

Comparative Analysis of Secondary Metabolites in *Diplodia corticola* Strains with Different Virulence Degrees associated with Canker and Dieback of *Quercus* spp.

Maria Michela Salvatore [1,2], Maria Teresa Russo [1], Rosario Nicoletti [3,4], Alla Eddine Mahamedi [5], Akila Berraf-Tebbal [6], Alessio Cimmino [1,7], Marco Masi [1,7], and Anna Andolfi [1,7*]

[1] Department of Chemical Sciences, University of Naples Federico II, 80126 Naples, Italy; mariamichela.salvatore@unina.it (M.M.S.); mariateresa.russo2@unina.it (M.T.R.); alessio.cimmino@unina.it (A.C.); marco.masi@unina.it (M.M.)
[2] Institute for Sustainable Plant Protection, National Research Council, 80055 Portici, Italy
[3] Department of Agriculture, University of Naples Federico II, 80055 Portici, Italy; rosario.nicoletti@crea.gov.it
[4] Council for Agricultural Research and Economics, Research Center for Olive, Fruit and Citrus Crops,
81100 Caserta, Italy
[5] Department of Biology, Faculty of Natural Sciences, Life and Earth Sciences, University of Ghardaïa, Ghardaïa 47000, Algeria; aladin1342@yahoo.com
[6] Mendeleum-Institute of Genetics and Plant Breeding, Faculty of Horticulture, Mendel University in Brno, 69144 Lednice, Czech Republic; berraf.a@hotmail.fr
[7] BAT Center-Interuniversity Center for Studies on Bioinspired Agro-Environmental Technology, University of Naples Federico II, 80055 Portici, Italy
* Correspondence: andolfi@unina.it

Abstract: *Diplodia corticola* is one of the most aggressive fungal pathogens of *Quercus* species involved in the decline of Mediterranean oak forests. In this study, three strains of *D. corticola* associated with holm (*Quercus ilex*) and cork (*Quercus suber*) oak trees exhibiting dieback symptoms and cankers in Algeria were selected to investigate the production of secondary metabolites. Metabolomic analyses revealed the production of several known compounds, such as sphaeropsidins, diplopyrones and diplofuranones. Moreover, the comparative investigation of secondary metabolites produced by the analyzed strains with different degrees of virulence revealed possible implications of these compounds in the fungal virulence. In particular, sphaeropsidins seem to be the main phytotoxic compounds of *D. corticola* involved in the infections of *Quercus* species, with a possible synergistic influence of the less representative compounds in the fungal virulence.

Keywords: culture filtrates; mycelial extracts; HPLC-ESI-HRMS; sphaeropsidins; quantitative analysis; virulence factors

Contents

Figure S1. ^{1}H NMR spectra of crude extracts obtained from culture filtrates (CE) of *Diplodia corticola* strains MAEC10, MAEC02, and MAEC03. CE10 (blue), CE02 (red), CE03 (green) recorded at 400 MHz in $CDCl_3$

Figure S2. ^{1}H NMR spectra of crude extracts obtained from mycelia (ME) of *Diplodia corticola* strains MAEC10, MAEC02, and MAEC03. ME10 (blue), ME02 (red), ME03 (green) recorded at 400 MHz in $CDCl_3$

Figure S3. Total Ion Chromatogram of crude extracts obtained from culture filtrates (CE) of *Diplodia corticola* strains MAEC10, MAEC02, and MAEC03. CE10 (blue), CE02 (red), CE03 (green)

Figure S4. Total Ion Chromatogram of crude extracts obtained from mycelia (ME) of *Diplodia corticola* strains MAEC10, MAEC02, and MAEC03. ME10 (blue), ME02 (red), ME03 (green)

Figure S5. HRESI MS (+) spectra of compounds detected in crude extracts obtained from culture filtrates (CE) and mycelia (ME) of strains of *Diplodia corticola*, MAEC 10, MAEC 02, MAEC 03. Precursor ions are reported for each compound in the main text.

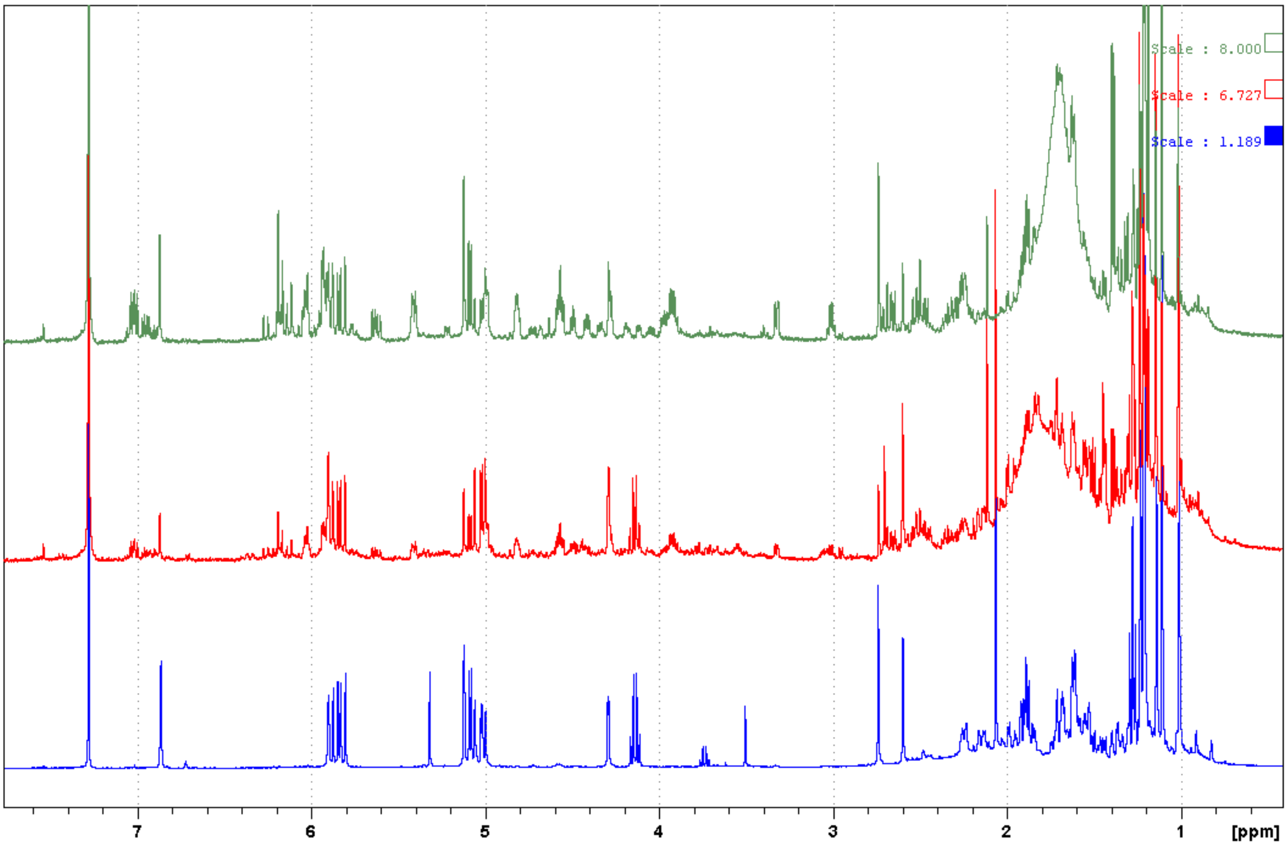

Figure S1. ^{1}H NMR spectra of crude extracts obtained from culture filtrates (CE) of *Diplodia corticola* strains MAEC10, MAEC02, and MAEC03. CE10 (blue), CE02 (red), CE03 (green) recorded at 400 MHz in $CDCl_3$

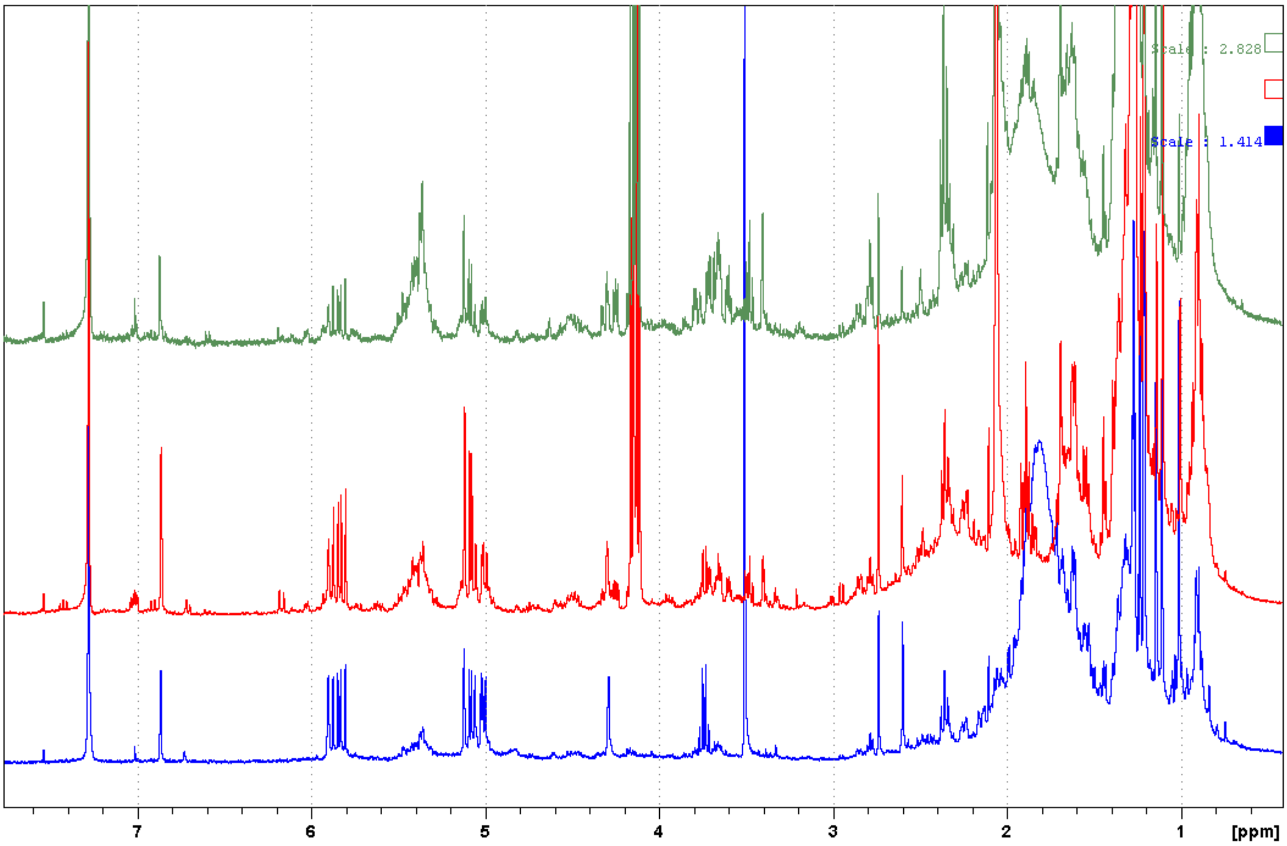

Figure S2. ^{1}H NMR spectra of crude extracts obtained from mycelia (ME) of *Diplodia corticola* strains MAEC10, MAEC02, and MAEC03. ME10 (blue), ME02 (red), ME03 (green) recorded at 400 MHz in $CDCl_3$

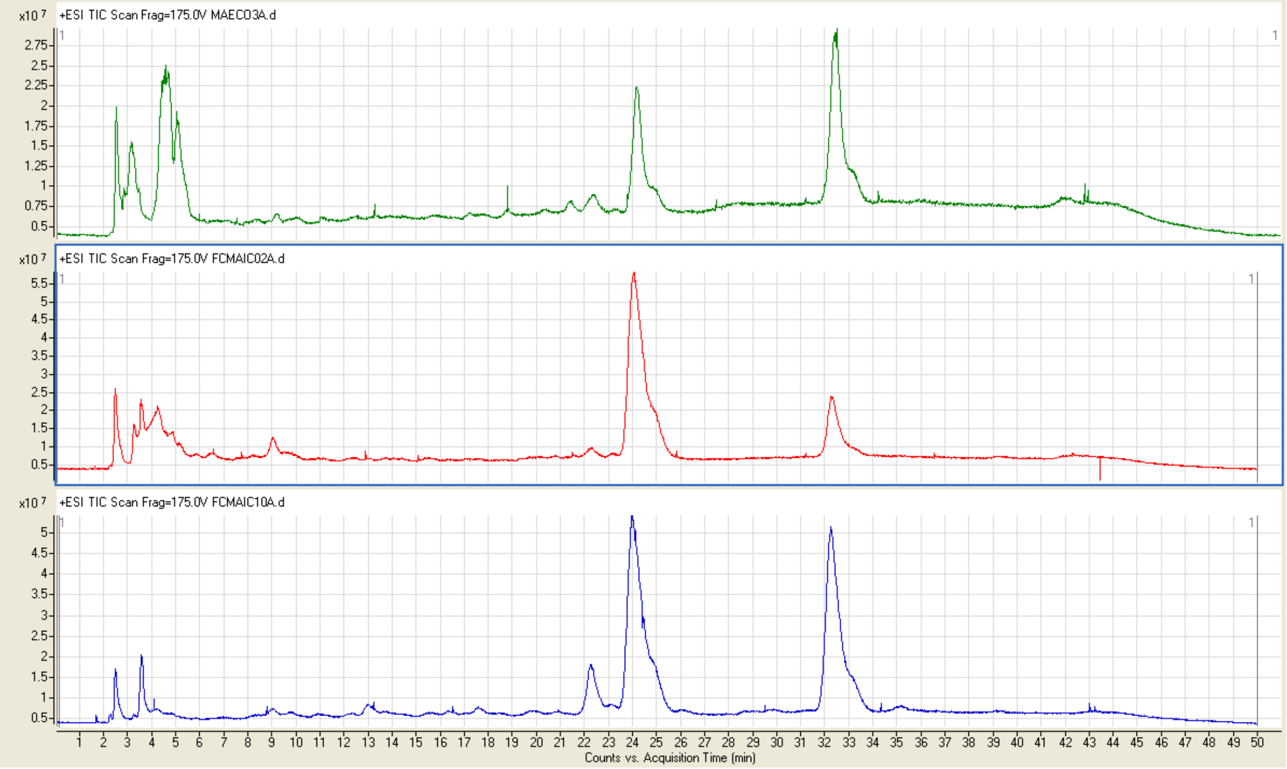

Figure S3. Total Ion Chromatogram of crude extracts obtained from culture filtrates (CE) of *Diplodia corticola* strains MAEC10, MAEC02, and MAEC03. CE10 (blue), CE02 (red), CE03 (green)

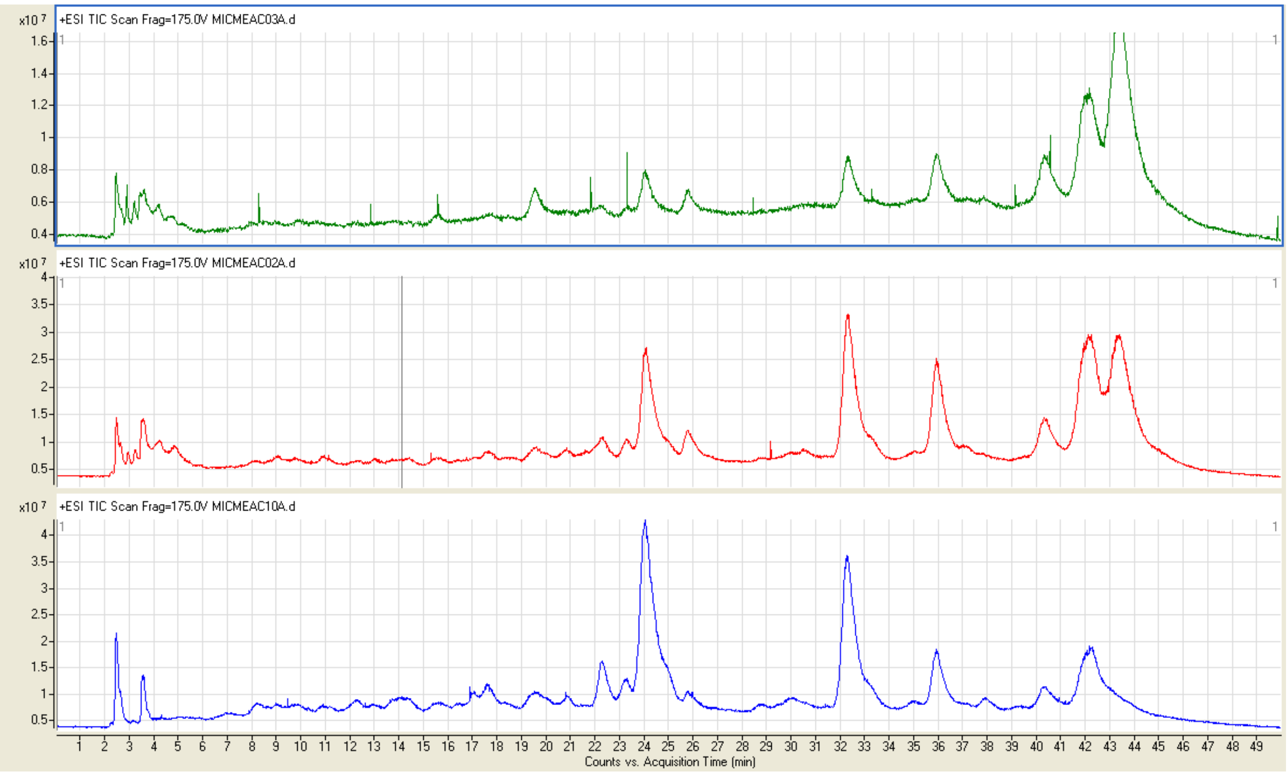

Figure S4. Total Ion Chromatogram of crude extracts obtained from mycelia (ME) of *Diplodia corticola* strains MAEC10, MAEC02, and MAEC03. ME10 (blue), ME02 (red), ME03 (green)

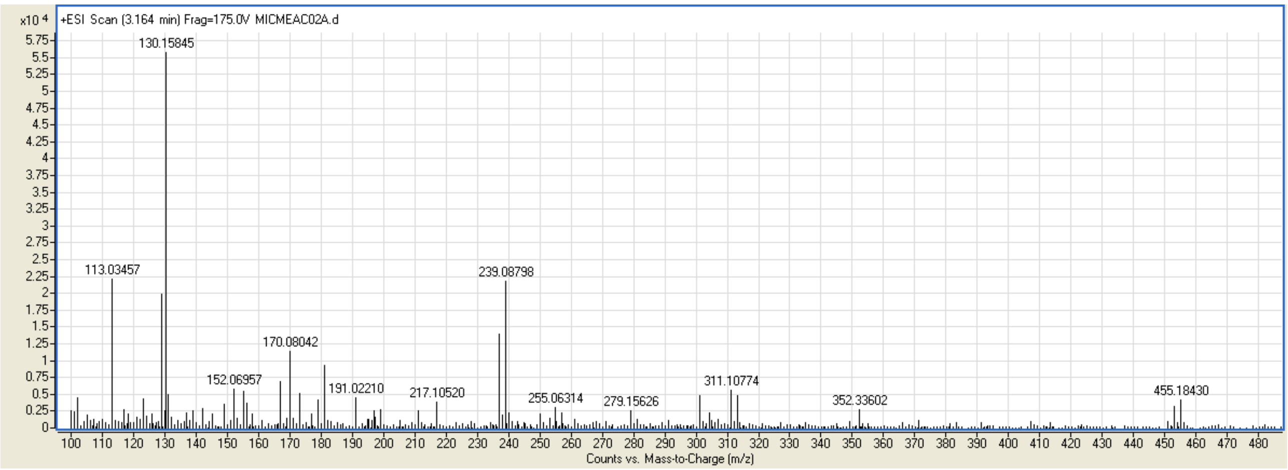

Unknown 1

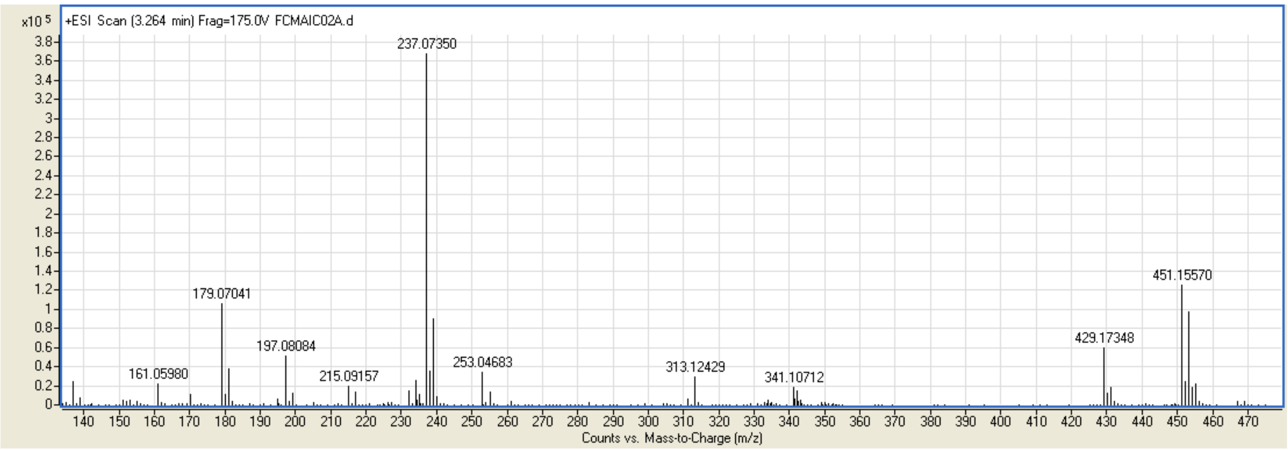

Unknown 2

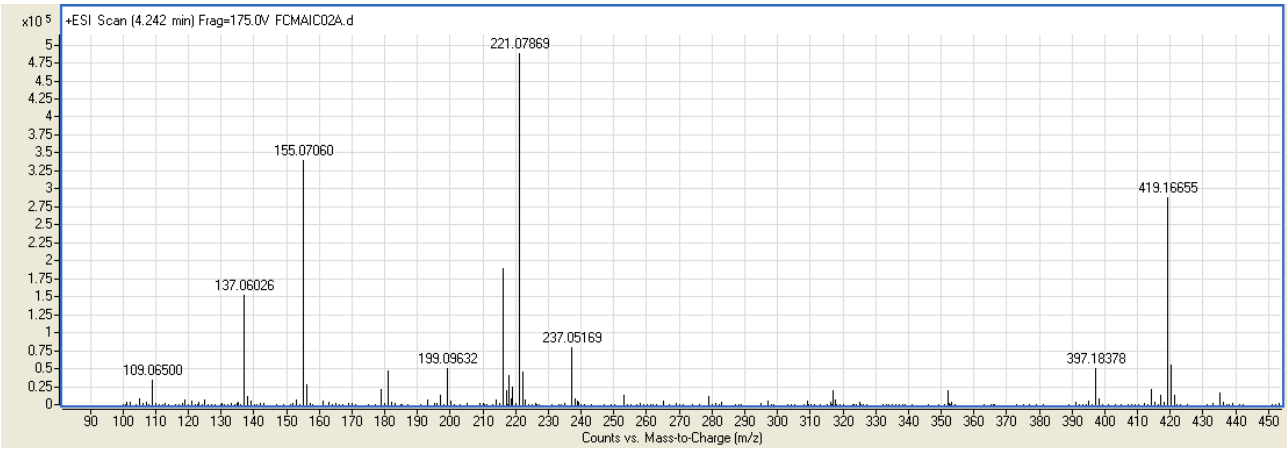

Diplobifuranylones A/B

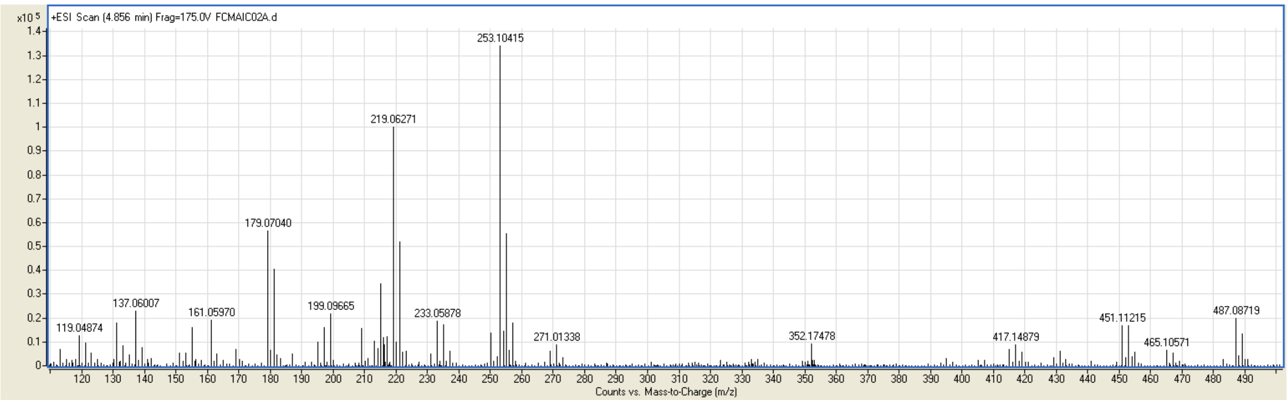

Diplopyrone C or diplobifuranylone C

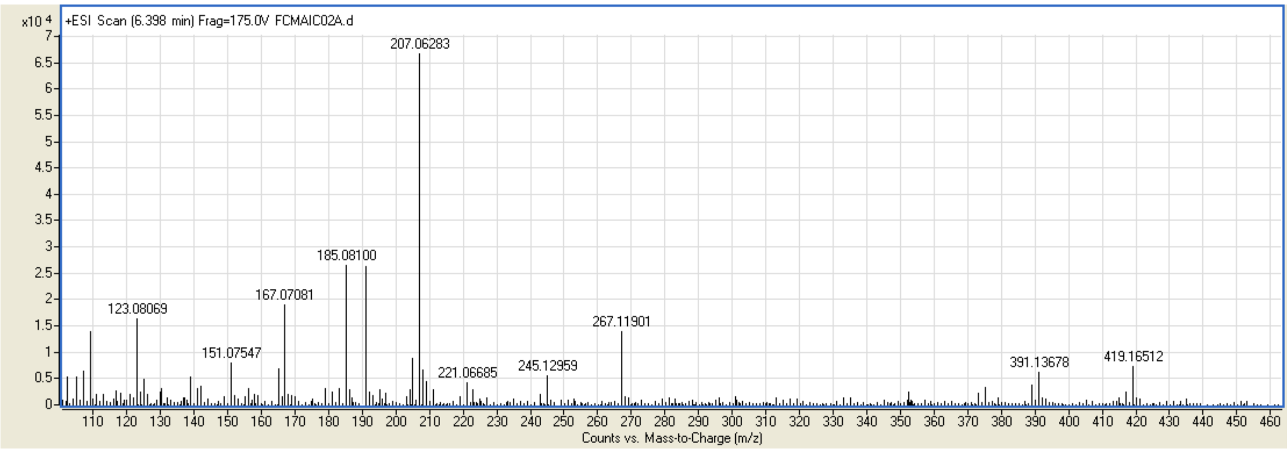

Pinofuranoxins A/B

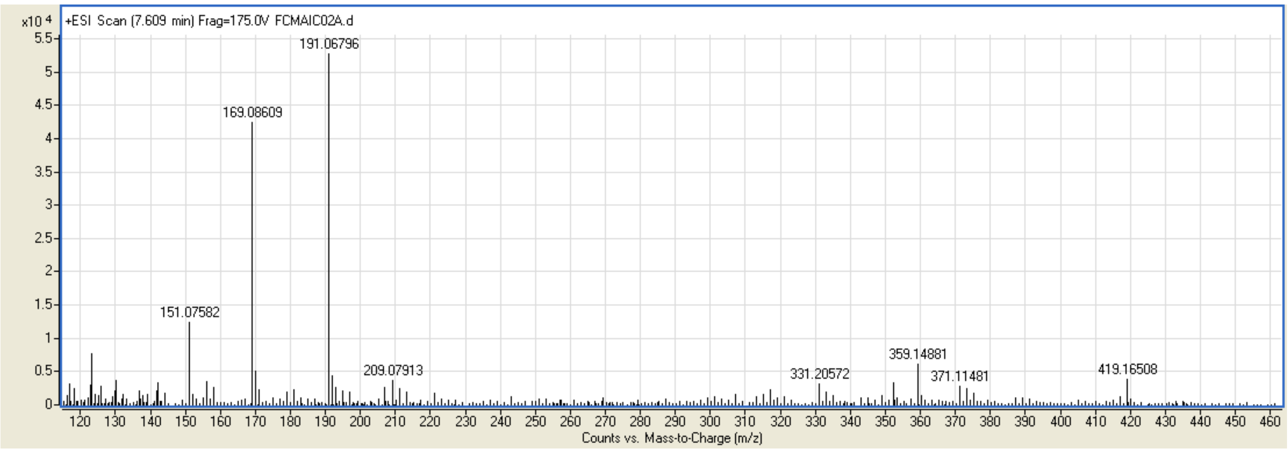

Unknown3

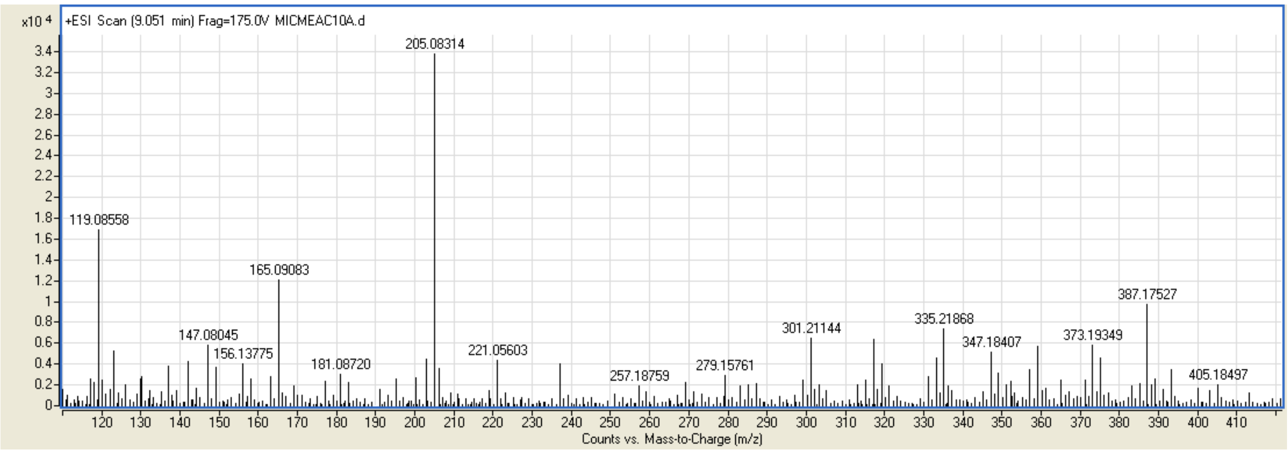

Diplofuranone A or Sapinofuranones A/B

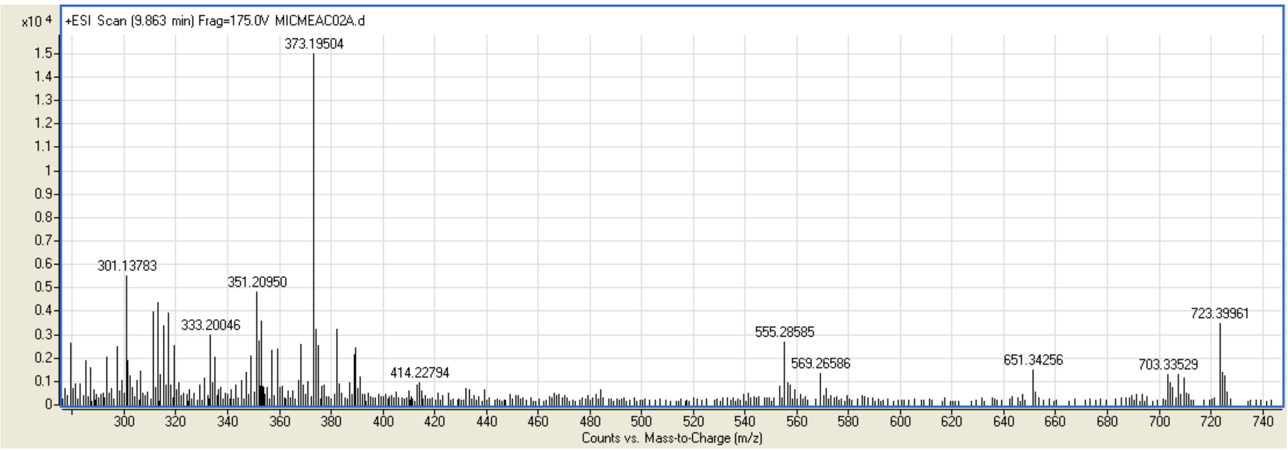

Unknown4

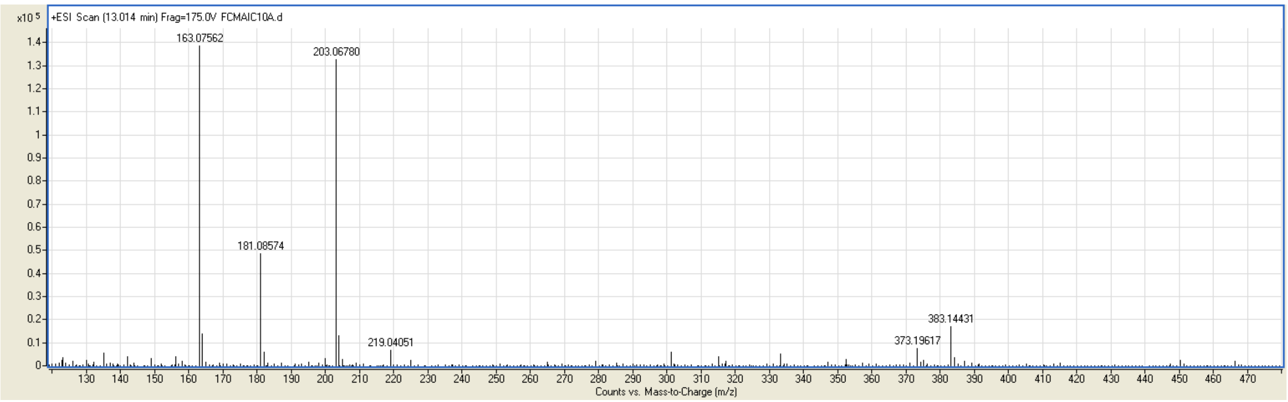

Diplopyrone B

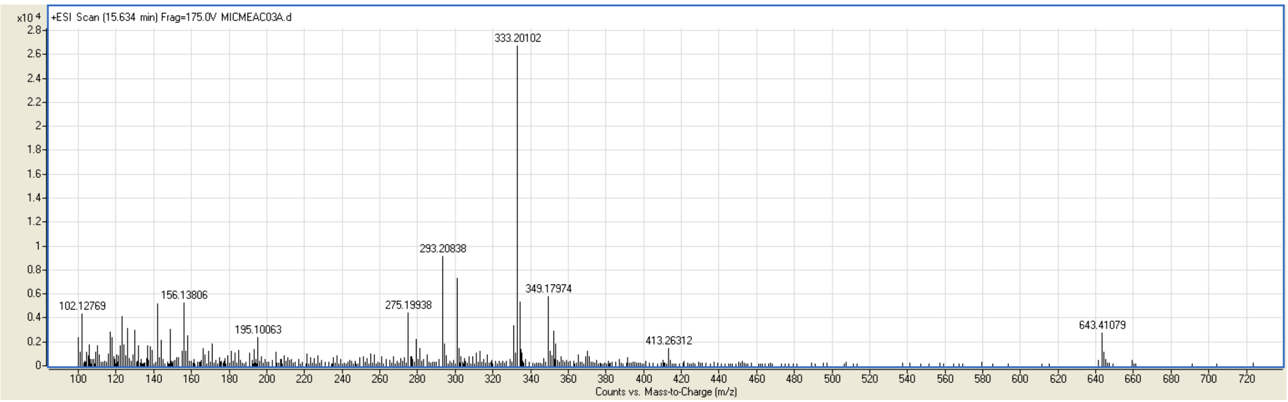

Unknown5

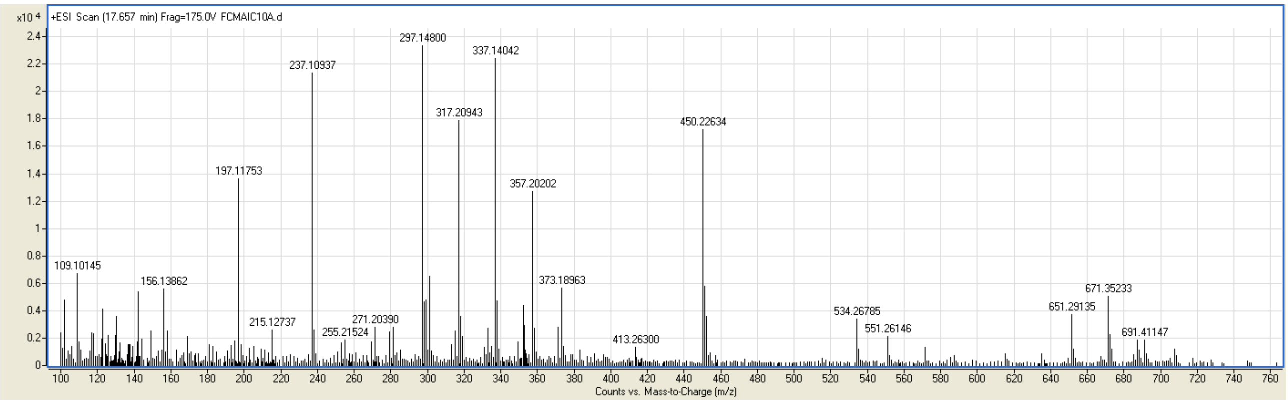

Unknown6

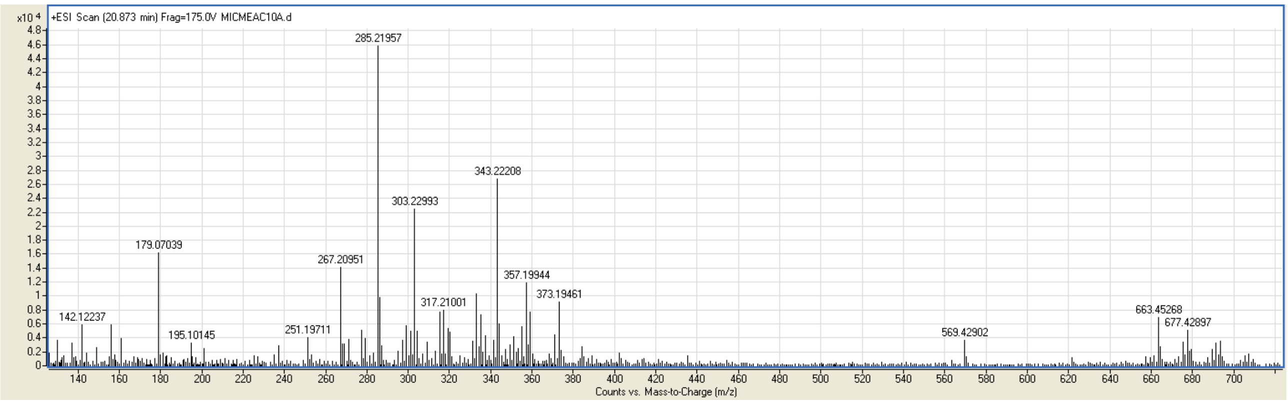

Unknown7

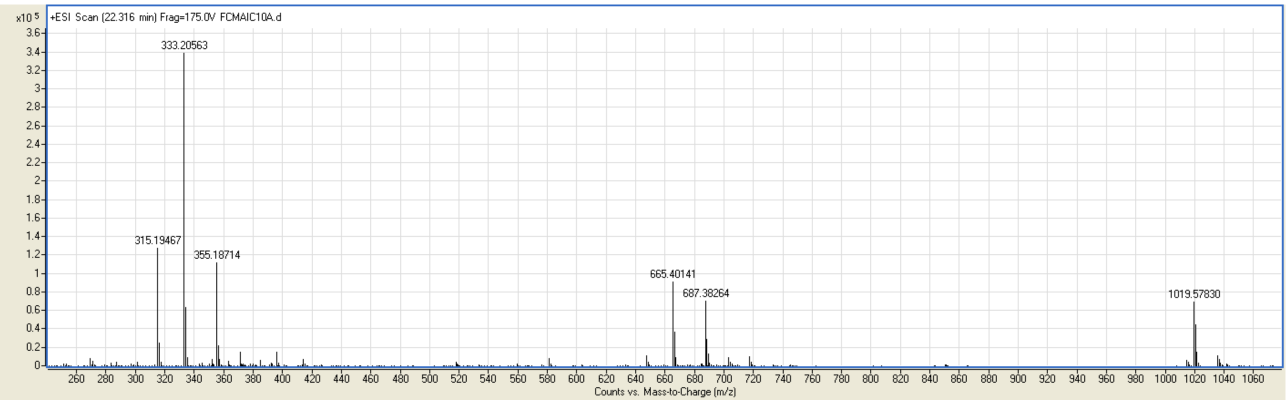

Sphaeropsidin C

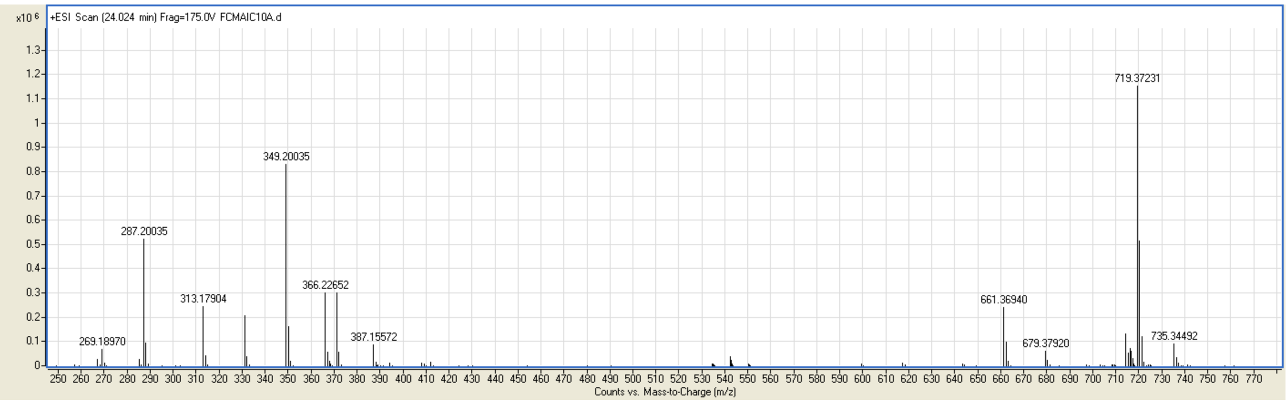

Sphaeropsidin B

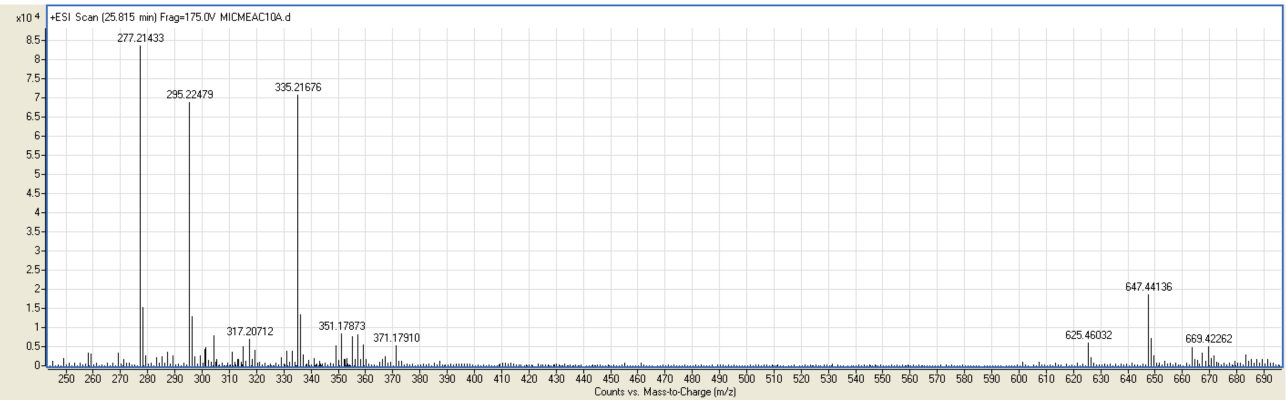

Unknown8

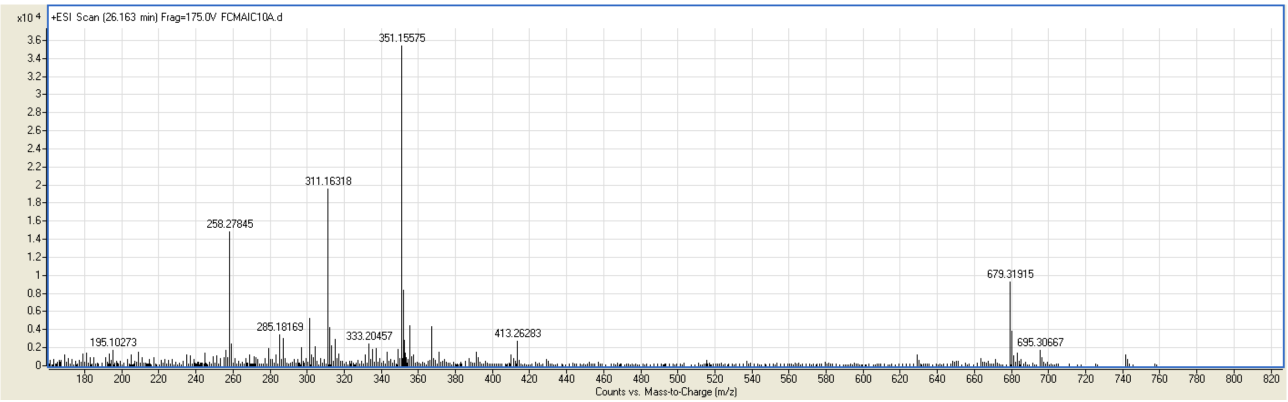

Unknown9

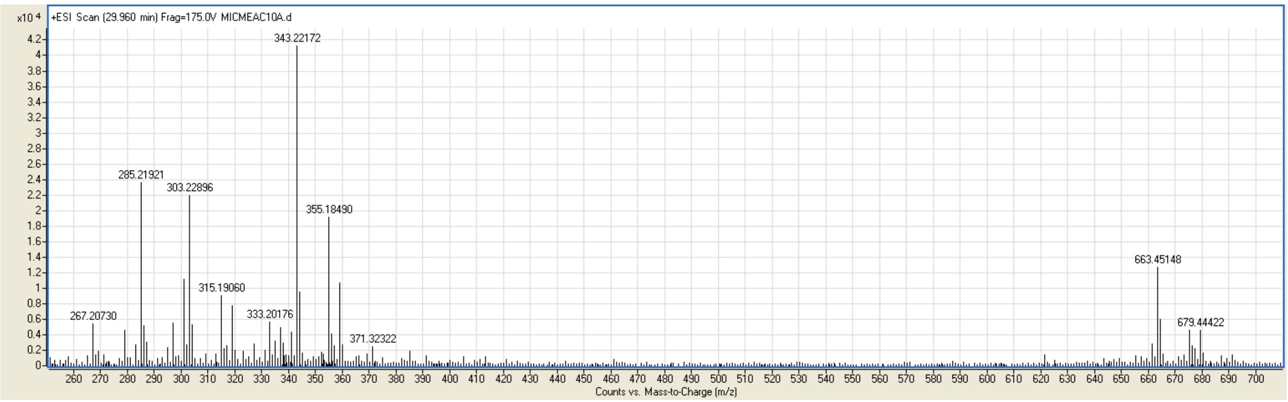

Unknown10

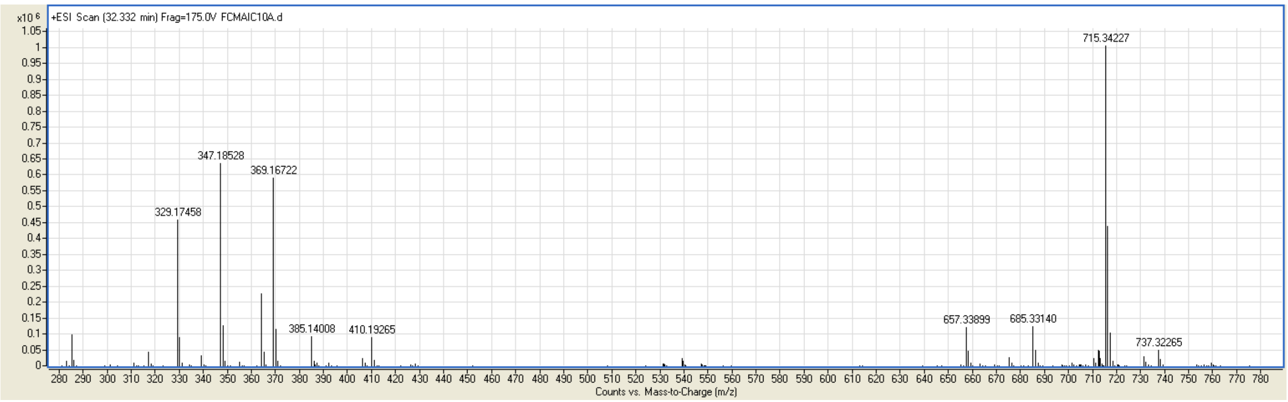

Sphaeropsidin A

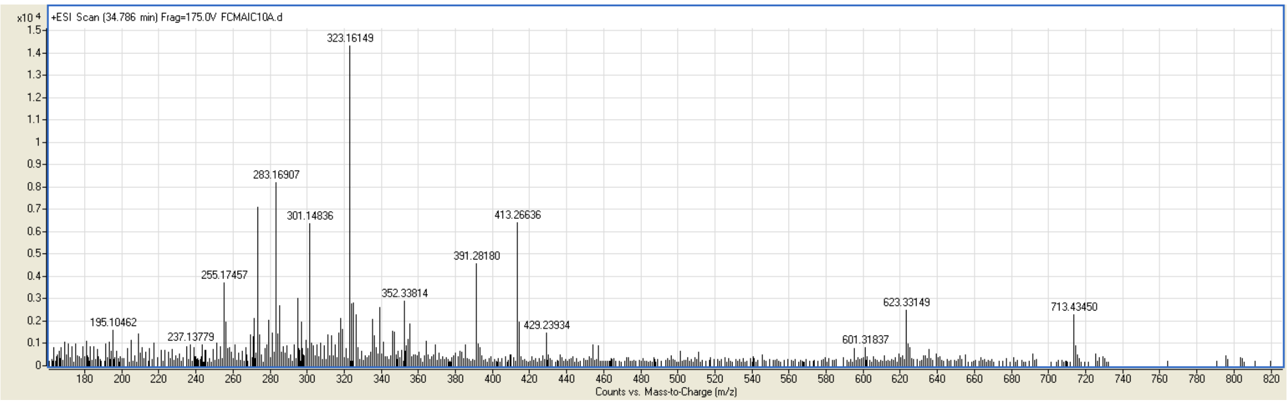

Unknown11

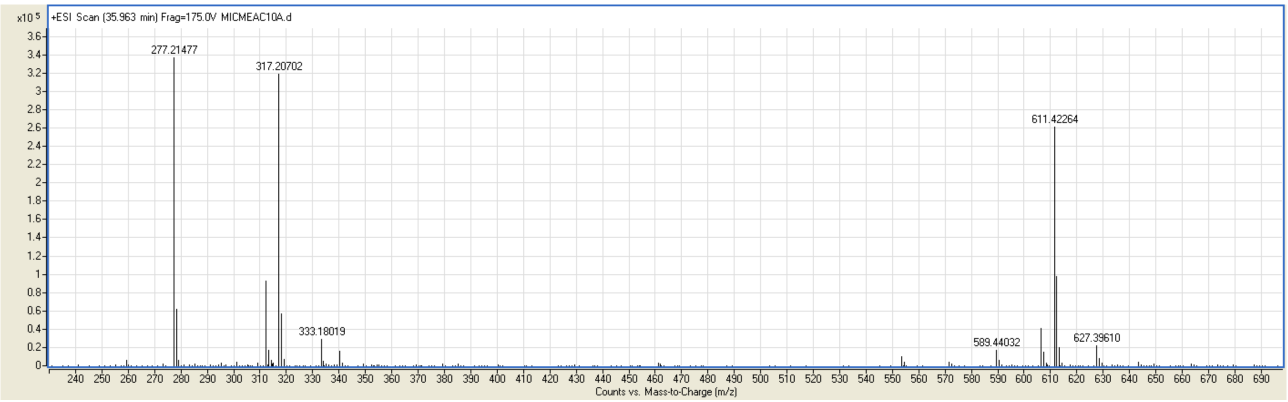

Hydroxylinolenic acid

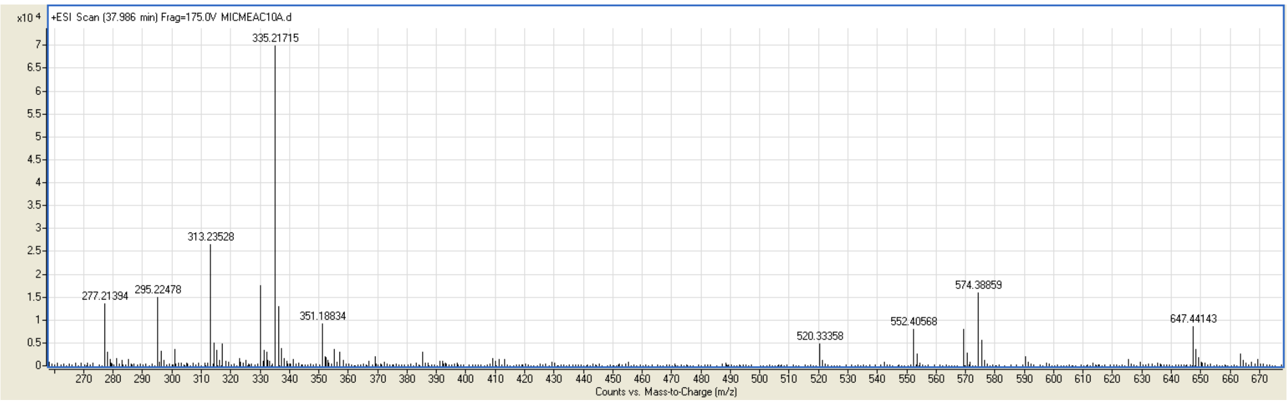

Unknown12

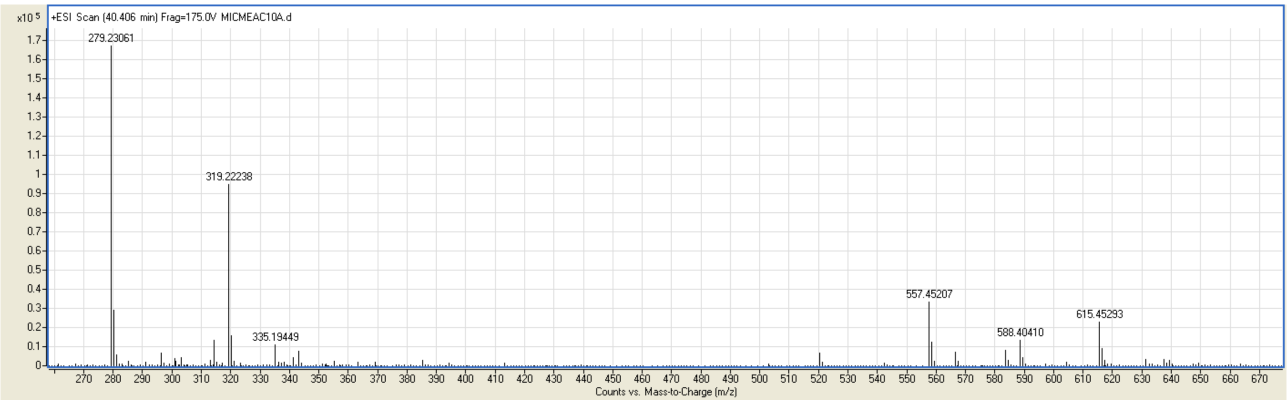

Hydroxylinoleic acid

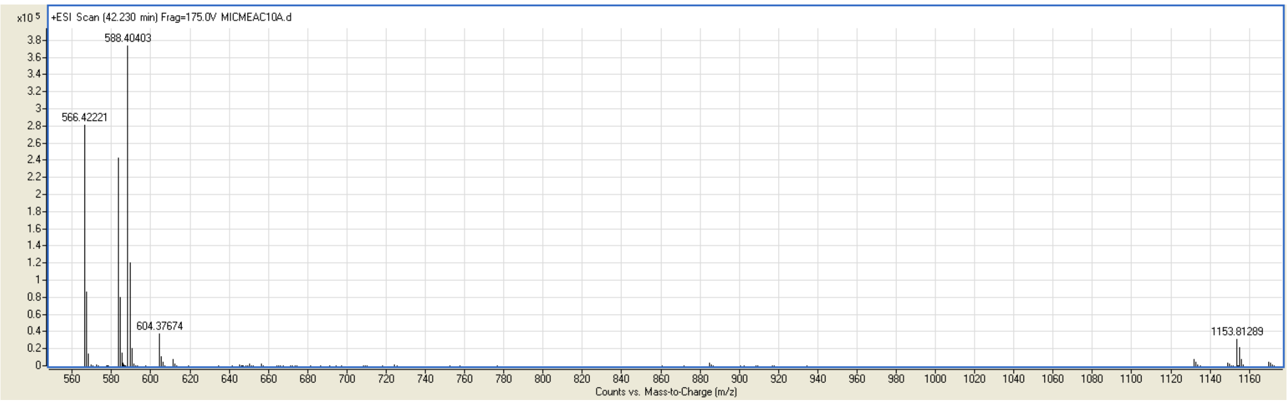

Unknown13

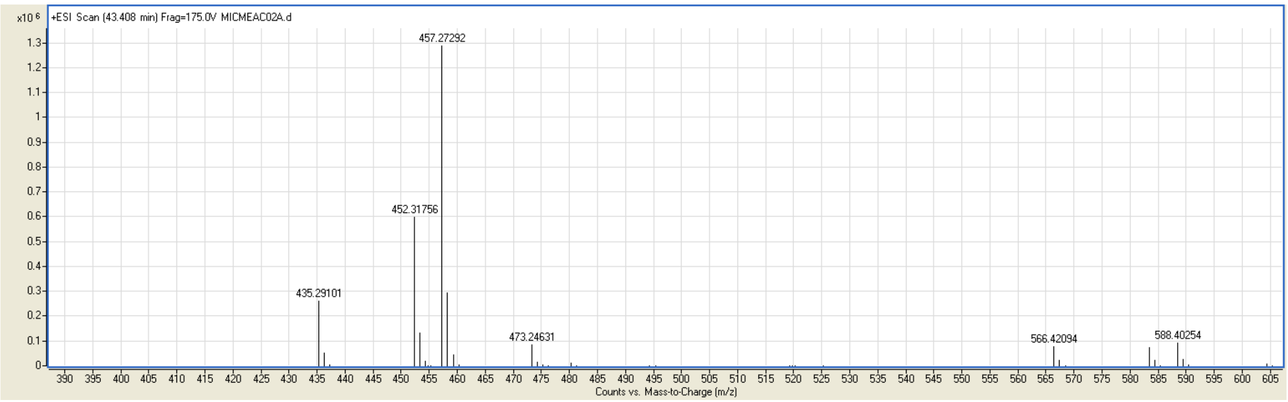

Unknown14

Figure S5. ESI-HRMS (+) spectra of compounds detected in crude extracts obtained from culture filtrates (CE) and mycelia (ME) of strains of *Diplodia corticola*, MAEC 10, MAEC 02, and MAEC 03. Precursor ions are reported for each compound in the main text.
